# Supplementary figures and images for: Transcription factor condensates, 3D clustering, and gene expression enhancement of the MET regulon
Source: eLife. 2024 Sep 30;13:RP96028. doi: 10.7554/eLife.96028 (PMC11441978; doi:10.7554/eLife.96028)

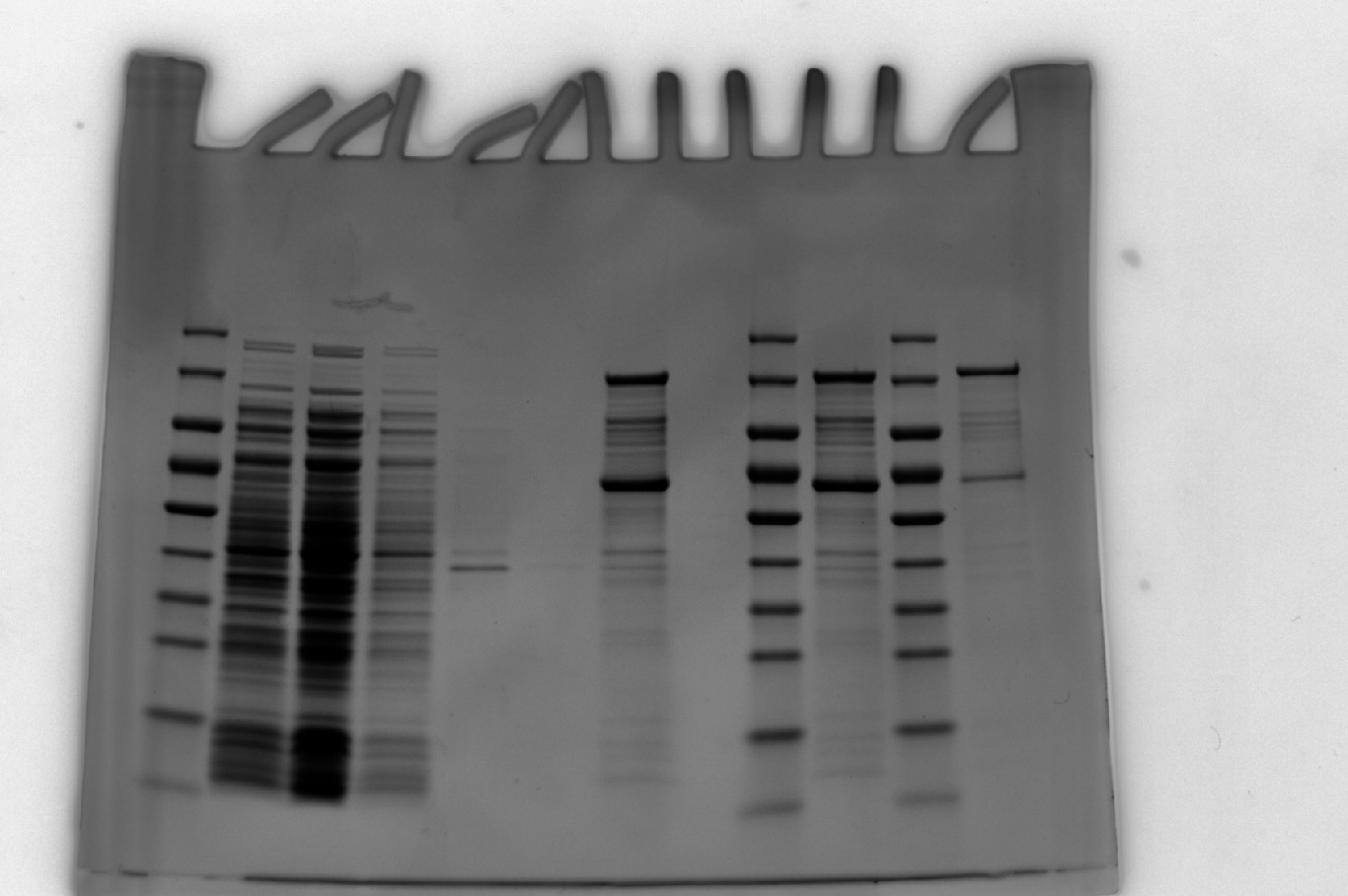

Supplement: Figure 2—source data 1. [file elife-96028-fig2-data1.zip › RawGelsBlots/Figure 2-source data 1. Raw Met4 Coomassie.jpg]

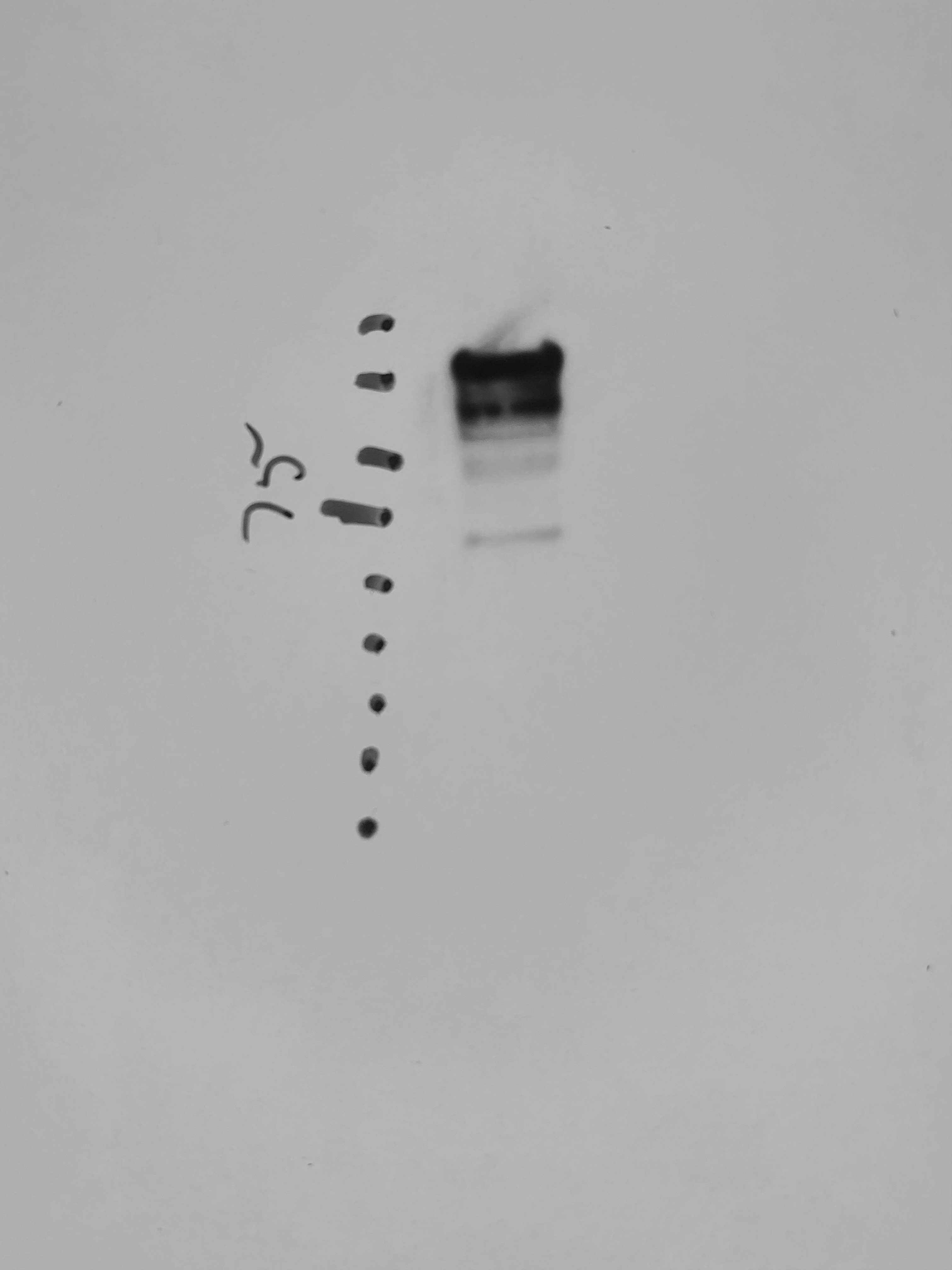

Supplement: Figure 2—source data 1. [file elife-96028-fig2-data1.zip › RawGelsBlots/Figure 2-source data 2. Raw Met4 anti-6xHis.jpeg]

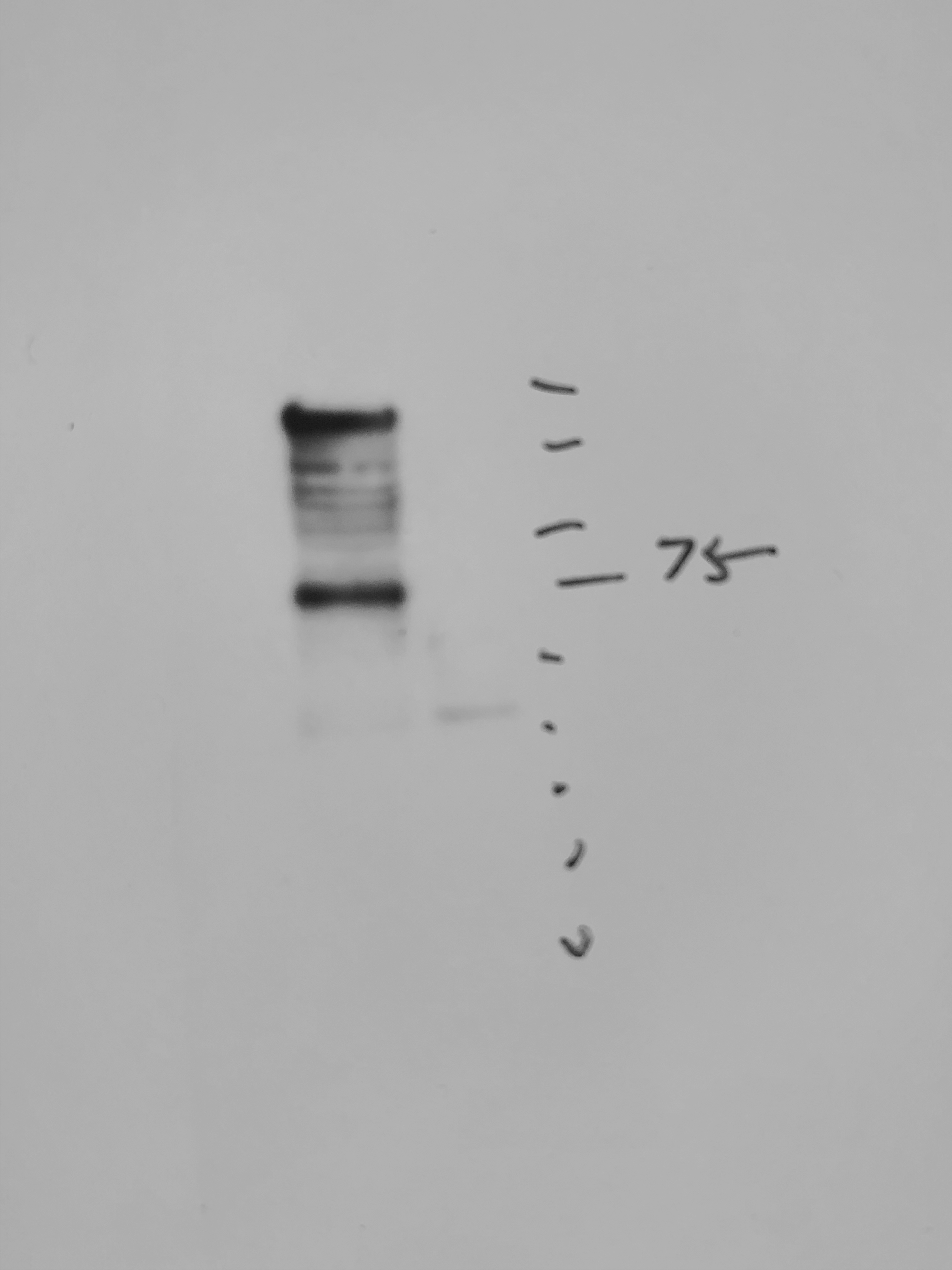

Supplement: Figure 2—source data 1. [file elife-96028-fig2-data1.zip › RawGelsBlots/Figure 2-source data 3. Raw Met4 anti-MBP.jpeg]

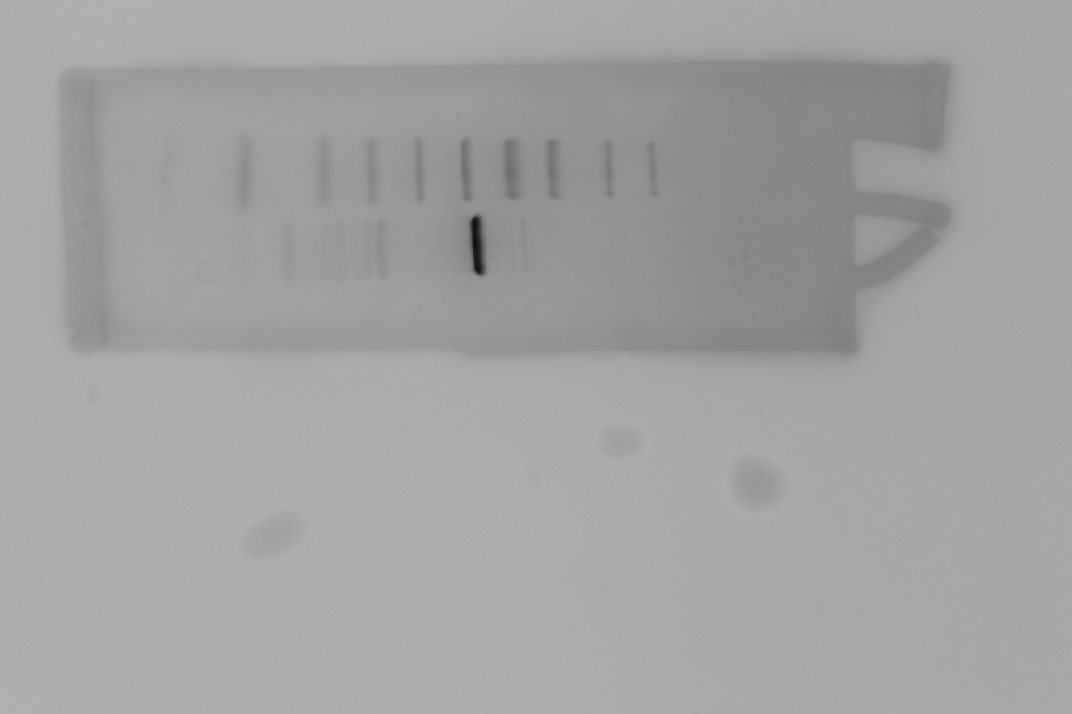

Supplement: Figure 2—source data 1. [file elife-96028-fig2-data1.zip › RawGelsBlots/Figure 2-source data 4. Raw Met32 Coomassie.jpg]

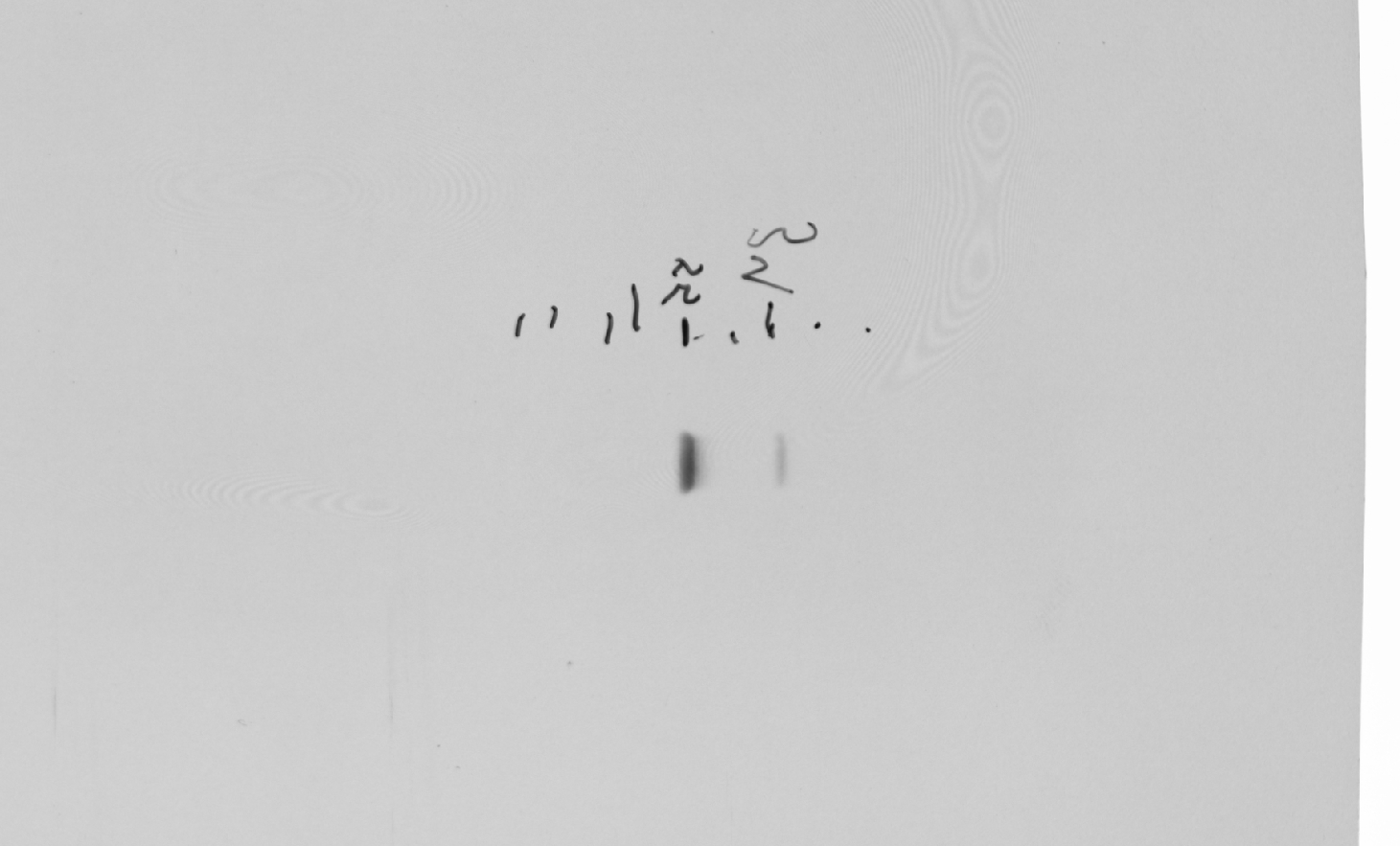

Supplement: Figure 2—source data 1. [file elife-96028-fig2-data1.zip › RawGelsBlots/Figure 2-source data 5. Raw Met32 anti-6xHis.jpg]

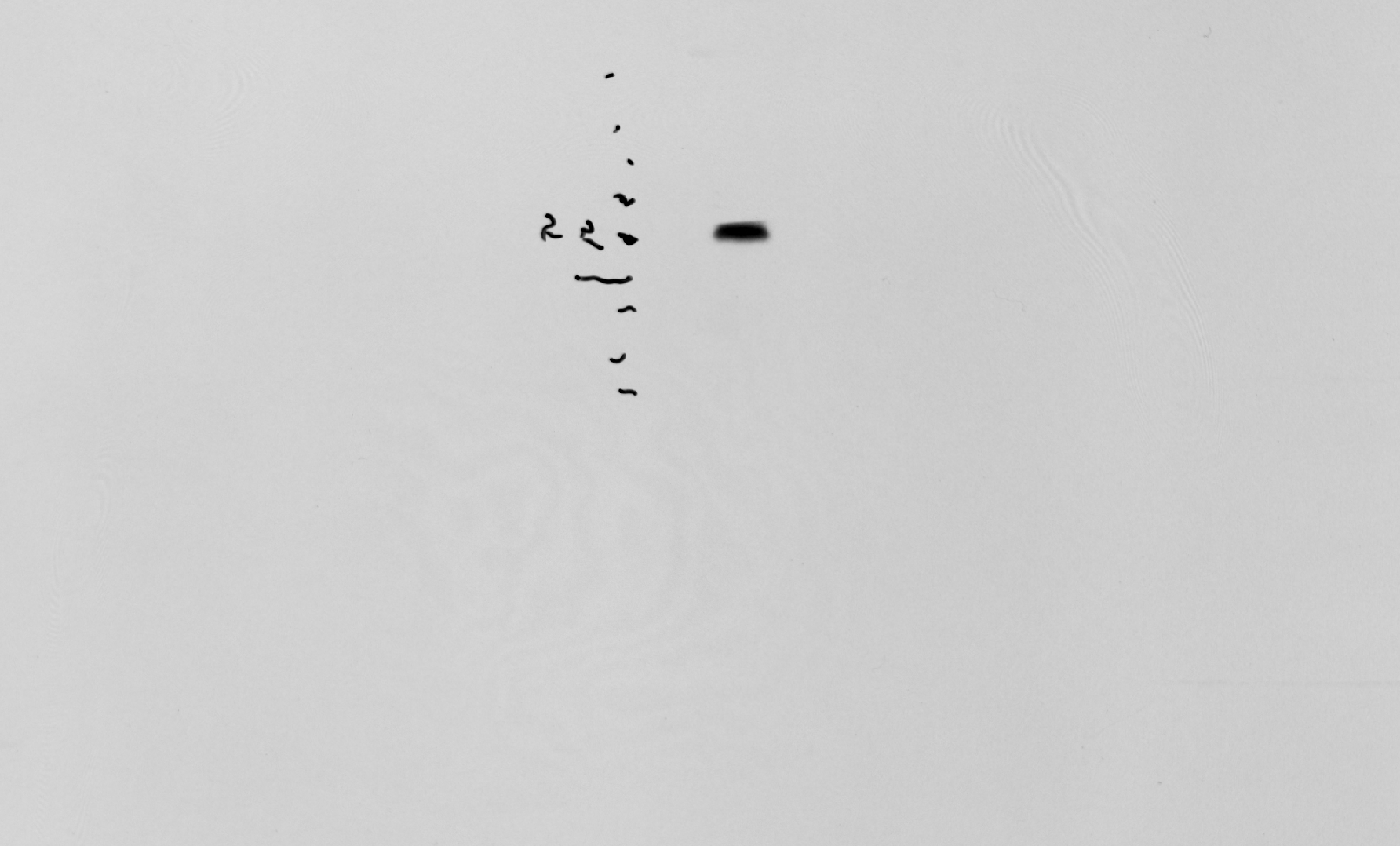

Supplement: Figure 2—source data 1. [file elife-96028-fig2-data1.zip › RawGelsBlots/Figure 2-source data 6. Raw Met32 anti-mCherry.jpg]

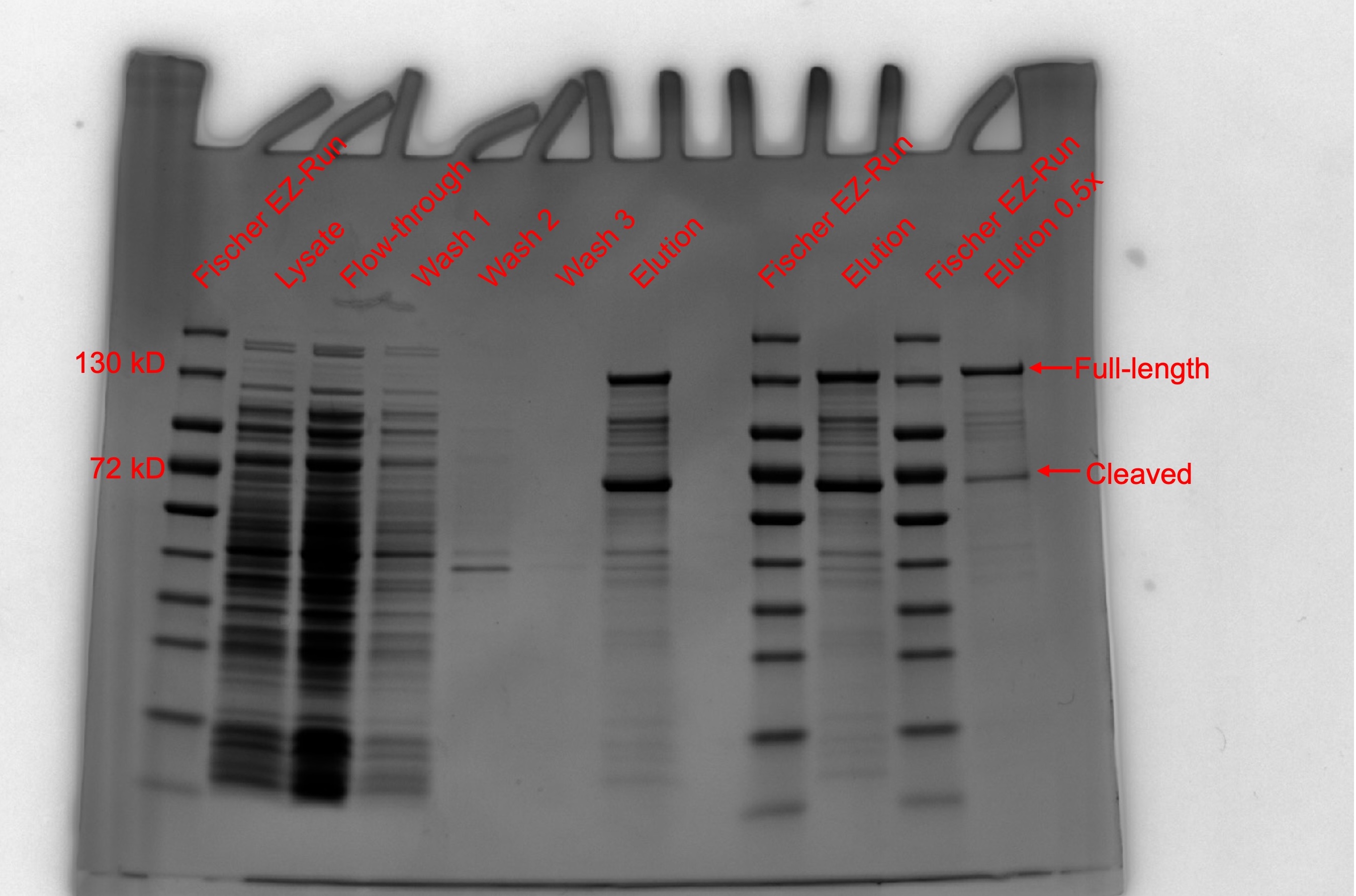

Supplement: Figure 2—source data 2. [file elife-96028-fig2-data2.zip › LabeledGelsBlots/Figure 2-source data 1. Labeled Met4 Coomassie.jpg]

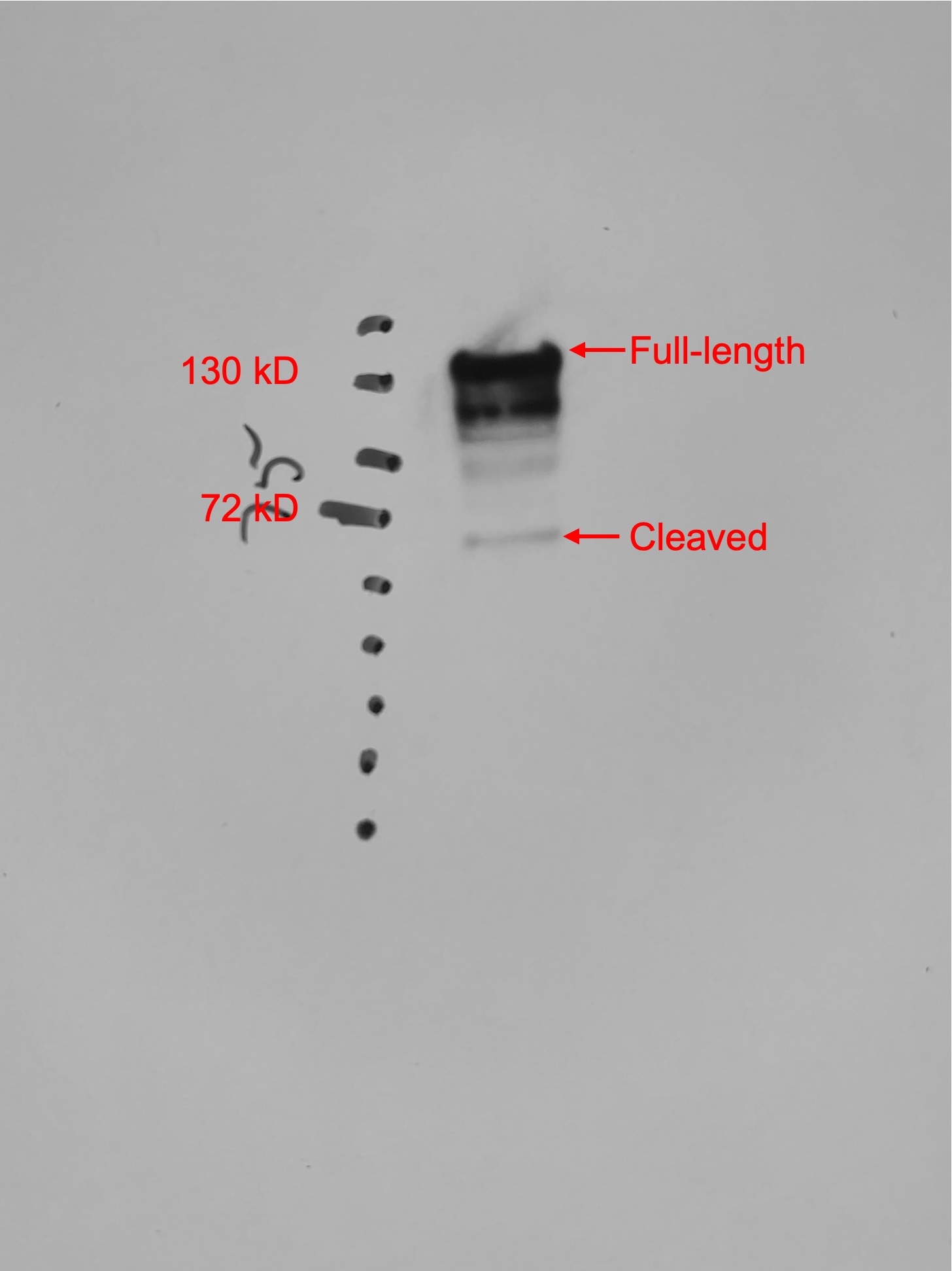

Supplement: Figure 2—source data 2. [file elife-96028-fig2-data2.zip › LabeledGelsBlots/Figure 2-source data 2. Labeled Met4 anti-6xHis.jpg]

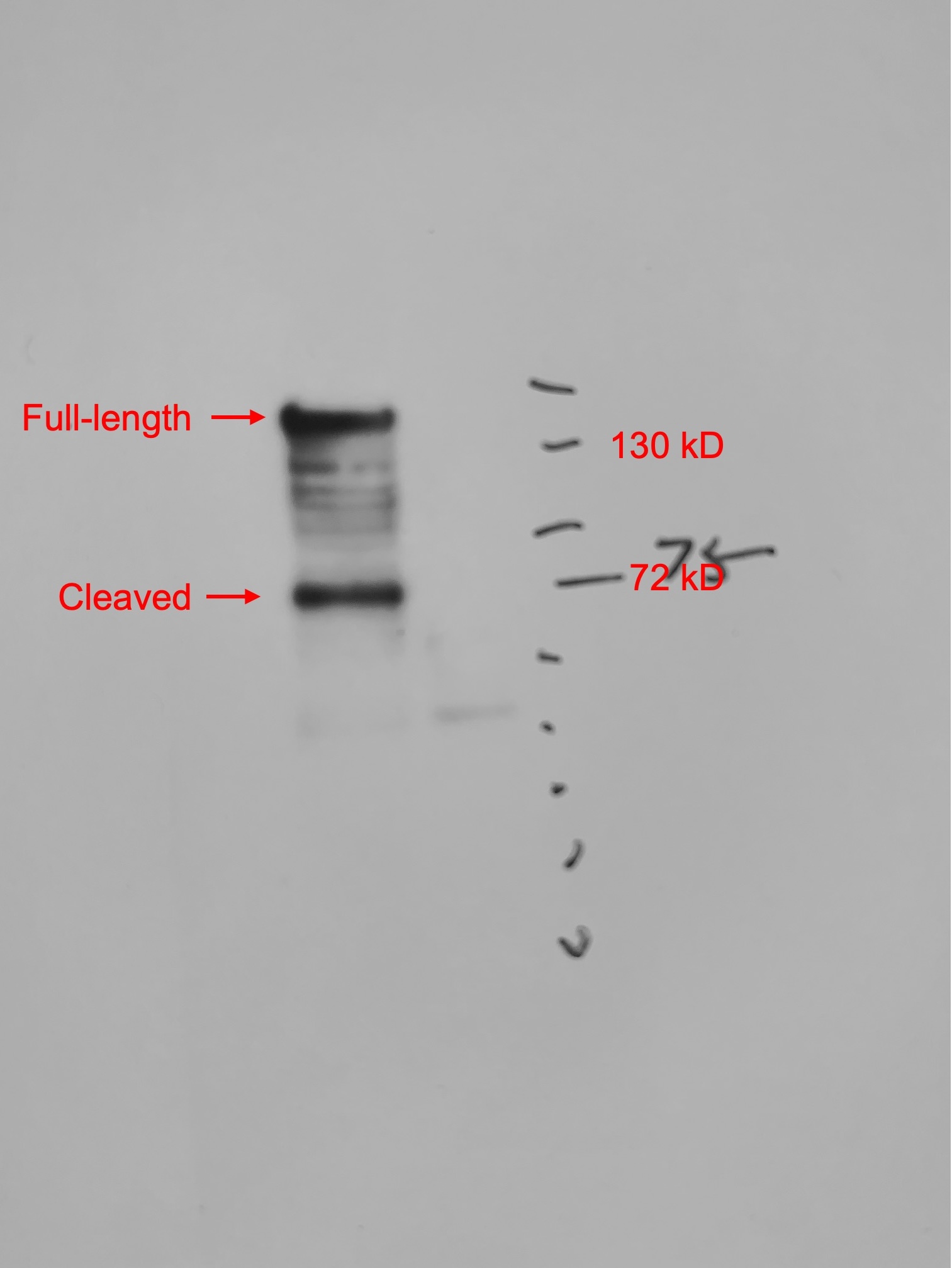

Supplement: Figure 2—source data 2. [file elife-96028-fig2-data2.zip › LabeledGelsBlots/Figure 2-source data 3. Labeled Met4 anti-MBP.jpg]

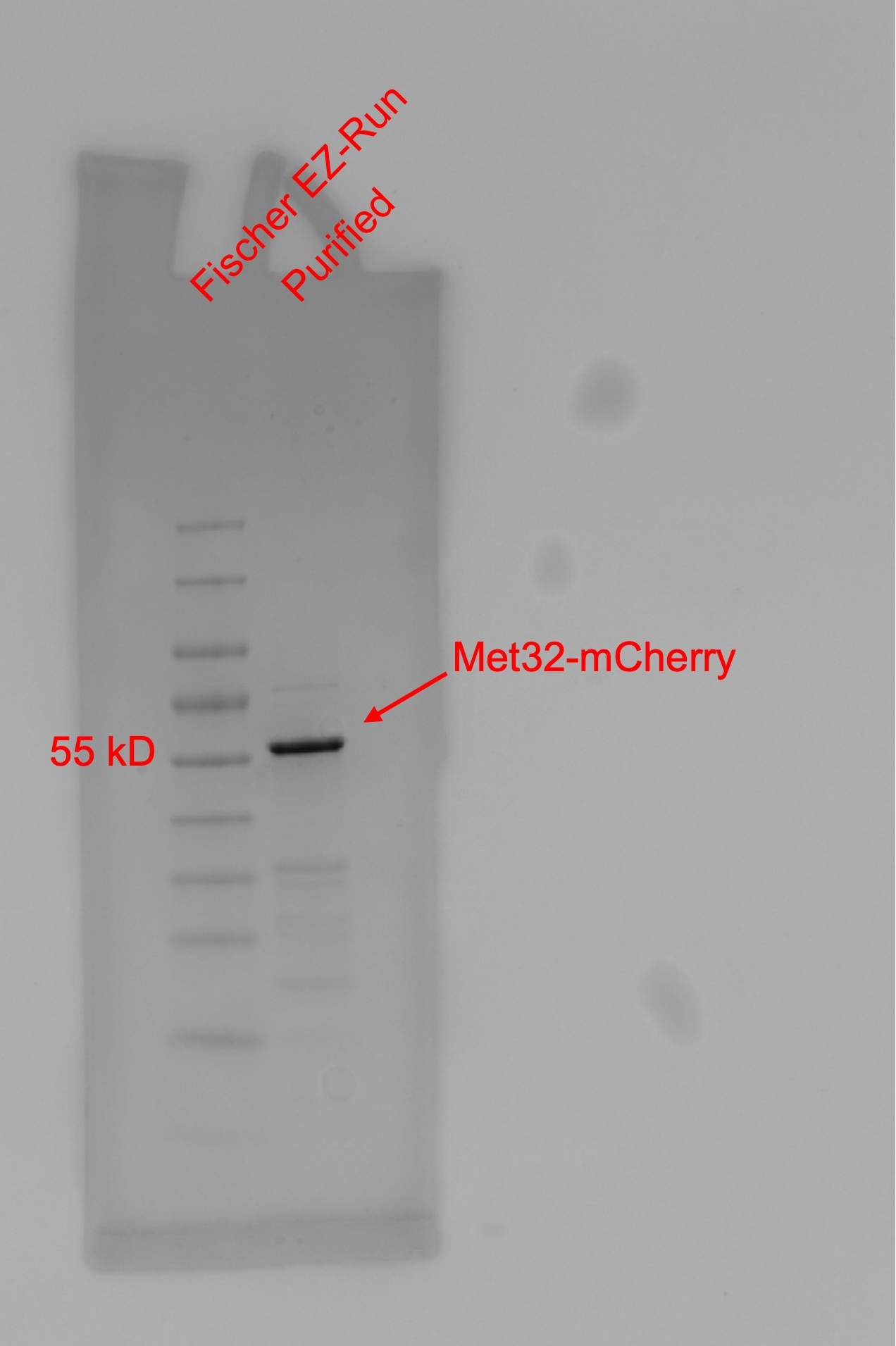

Supplement: Figure 2—source data 2. [file elife-96028-fig2-data2.zip › LabeledGelsBlots/Figure 2-source data 4. Labeled Met32 Coomassie.jpg]

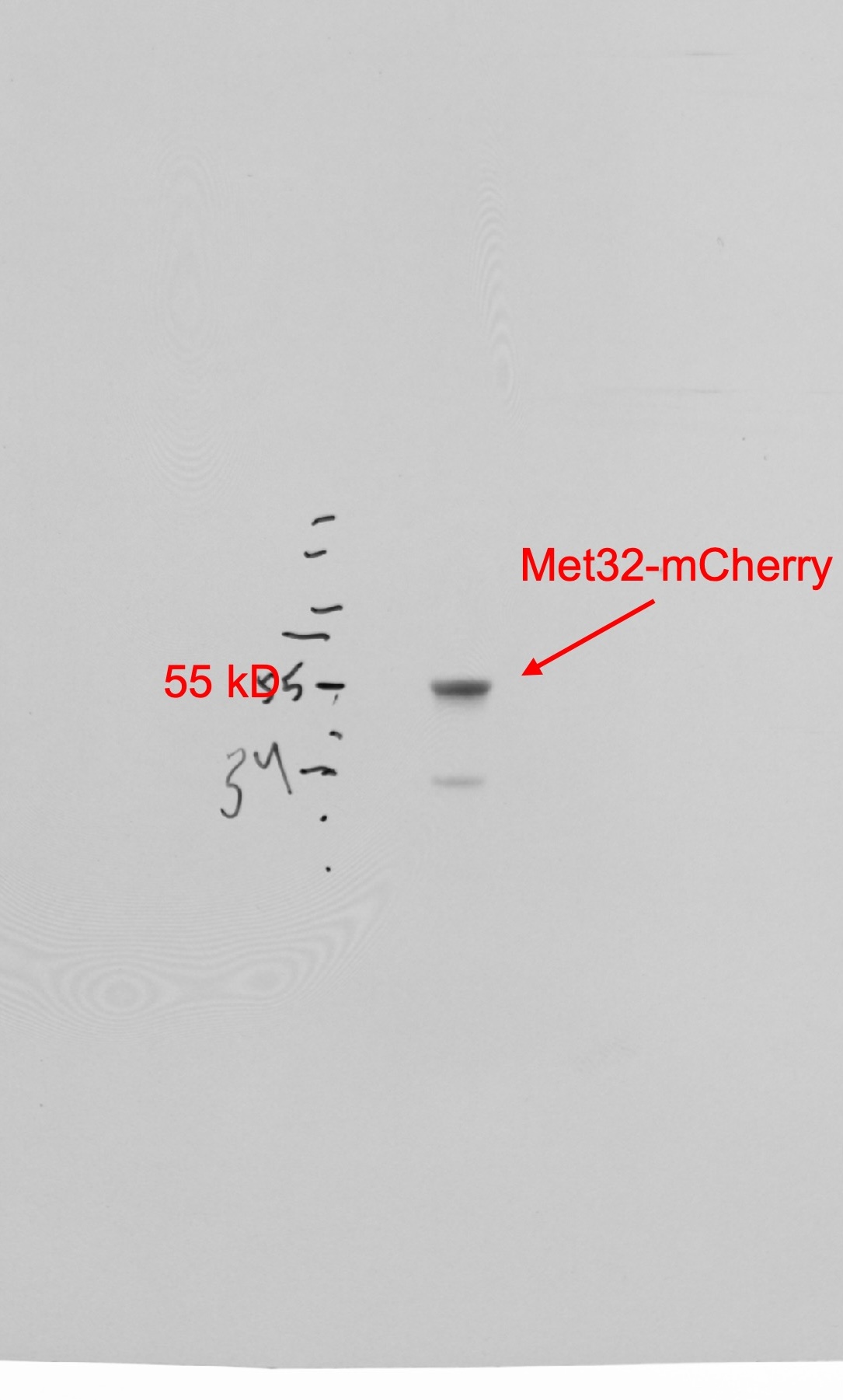

Supplement: Figure 2—source data 2. [file elife-96028-fig2-data2.zip › LabeledGelsBlots/Figure 2-source data 5. Labeled Met32 anti-6xHis.jpg]

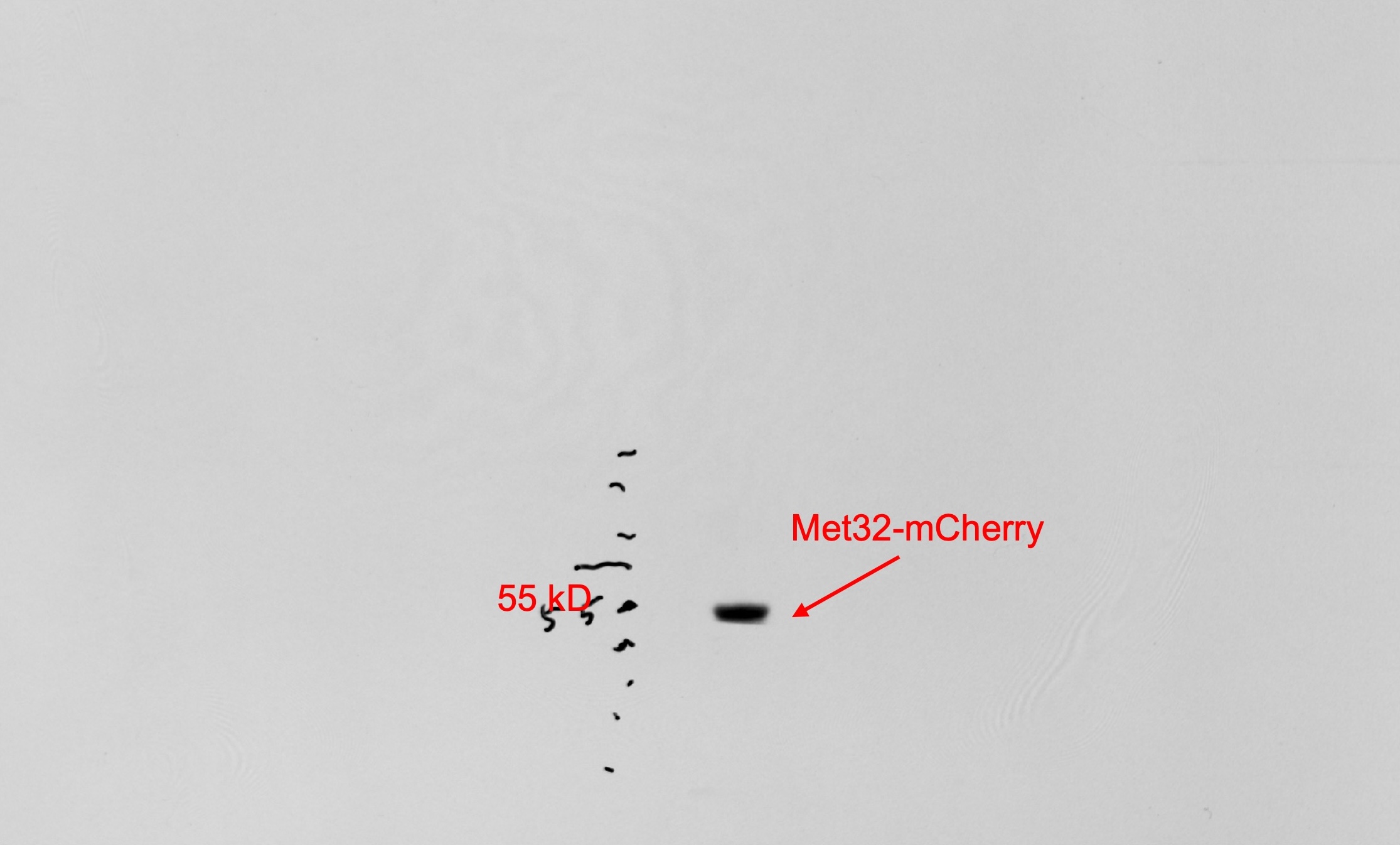

Supplement: Figure 2—source data 2. [file elife-96028-fig2-data2.zip › LabeledGelsBlots/Figure 2-source data 6. Labeled Met32 anti-mCherry.jpg]

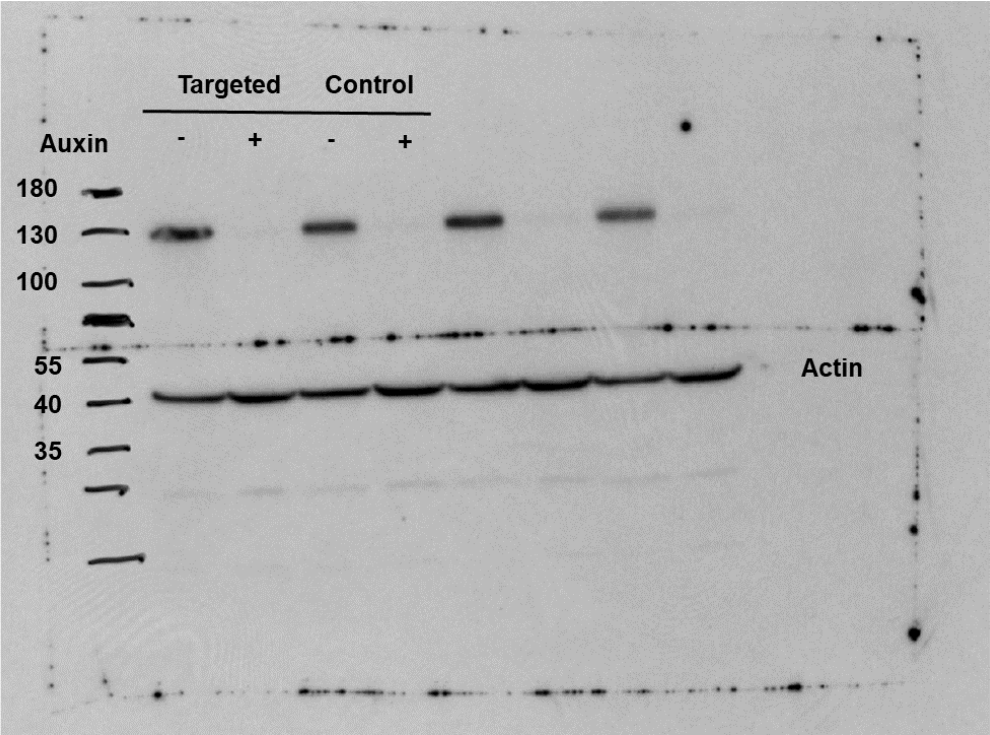

Supplement: Figure 4—figure supplement 1—source data 1. [file elife-96028-fig4-figsupp1-data1.zip › Figure4-figure supplement 1-source data 1/Figure4-figure supplement 1-source data 1. Uncropped and labelled gels for Figure4-figure supplement 1.pdf]

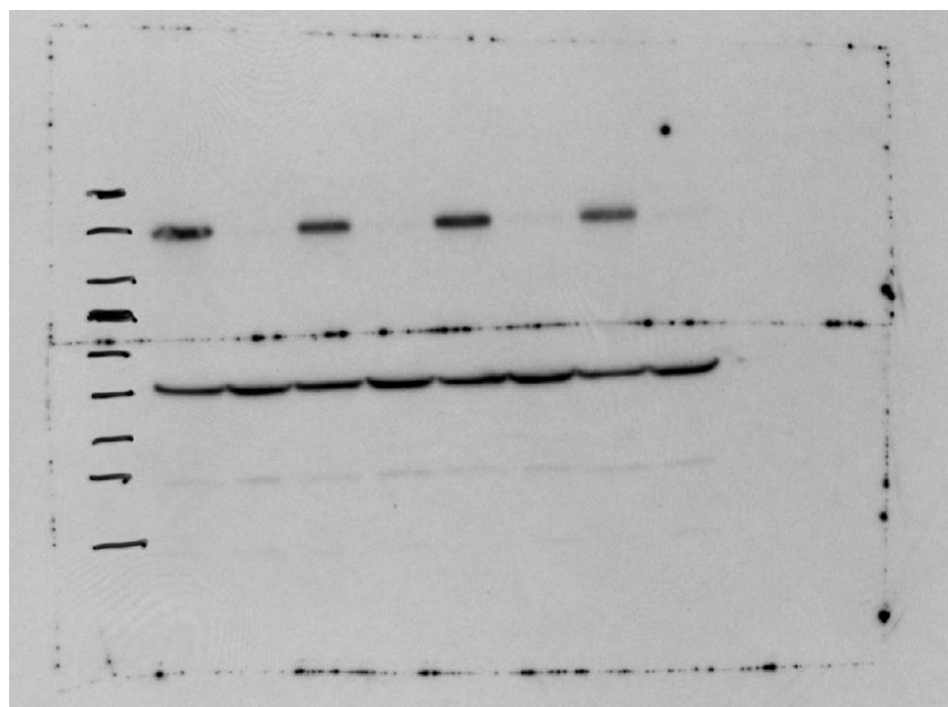

Supplement: Figure 4—figure supplement 1—source data 2. [file elife-96028-fig4-figsupp1-data2.zip › Figure4-figure supplement 1-source data 2/Figure4-figure supplement 1-source data 2. Raw unedited gels for Figure4-figure supplement 1.pdf]
